# Nutritional and Anti-Nutritional Factors in *Vicia sativa* L. Seeds and the Variability of Phenotypic and Morphological Characteristics of Some Vetch Accessions Cultivated in European Countries

**DOI:** 10.3390/ani11010044

**Published:** 2020-12-28

**Authors:** Eugeniusz R. Grela, Wioletta Samolińska, Wojciech Rybiński, Bożena Kiczorowska, Edyta Kowalczuk-Vasilev, Jan Matras, Sylwia Wesołowska

**Affiliations:** 1 Institute of Animal Nutrition and Bromatology, University of Life Sciences, 20-934 Lublin, Poland; eugeniusz.grela@up.lublin.pl (E.R.G.); bozena.kiczorowska@up.lublin.pl (B.K.); edyta.kowalczuk@up.lublin.pl (E.K.-V.); jan.matars@up.lublin.pl (J.M.); 2 Institute of Plant Genetics, Polish Academy of Sciences, 60-479 Poznań, Poland; wryb@igr.poznan.pl; 3 Institute of Soil Science and Environment Shaping, University of Life Sciences, 20-069 Lublin, Poland; sylwia.wesolowska@up.lublin.pl

**Keywords:** *Vicia sativa*, phenotypic traits, morphological traits, nutrients, anti-nutritional factors

## Abstract

**Simple Summary:**

This study was designed to determine the contents of nutrients and some anti-nutritional factors in the seeds of common vetch (*Vicia sativa* L.) and the variation of phenotypic and morphological traits in field studies of 44 European accessions, originating from Russia, Ukraine, Poland, the Czech Republic, the Slovak Republic, Hungary, and Germany and grown in the same soil-climate conditions. The results of the research showed that common vetch seeds may be valuable protein-rich feed stuff, but their nutritional usefulness is limited by the presence of anti-nutritional factors.

**Abstract:**

Agricultural research has traditionally focused on staple crops, while relatively little attention has been given to minor crops. Therefore, this study aimed to determine the nutrient contents and some anti-nutritional factors in the seeds of common vetch (*Vicia sativa* L.) and the variation of phenotypic and morphological traits in field studies of 44 European accessions, originating from Russia, Ukraine, Poland (east and east-central Europe), the Czech Republic, the Slovak Republic, Hungary (south-central Europe), and Germany (west-central Europe) and grown in the same soil-climate conditions. A three-year field study was conducted from 2010 to 2012. Accessions originating from west-central Europe flowered three days earlier than those from south-central Europe. They also had the lowest seed number per pod (5.9) but the highest thousand seed weight (58 g) (*p* < 0.05). Vetch lines coming from south-central Europe contained the highest level of crude protein in comparison with vetch seeds originating from west-central Europe (353 vs. 324 g kg^−1^, respectively) (*p* < 0.05), but the latter had the highest essential amino acid index value (75 vs. 71 in west-central Europe and south-central Europe, respectively) (*p* < 0.05). The highest protein level was noted in the seeds of Slovak origin (average 358 g kg^−1^), whereas the lowest protein level (324 g kg^−1^) was determined in the Russian and German lines. Vetch grain fat was rich in linoleic (53%) and linolenic (14%) acids. The best n-6/n-3 fatty acid ratio (4) and the highest α-linolenic acid level were exhibited by the Slovak and Polish accessions (*p* < 0.05). The seeds of vetch accessions from east, east-central, and south-central Europe contained higher levels of (*p* < 0.05) iron than those originating from west-central Europe. The concentration of tannins, trypsin inhibitors, and hydrogen cyanide reached on average 8, 3, and 81 mg kg^−1^, respectively. The highest hydrogen cyanide (HCN) levels was determined in the accessions of German and Russian origin (126 and 119 mg kg^−1^, respectively), and the lowest values were noted in the Slovak and Polish lines (50 and 67 mg HCN kg^−1^, respectively). Furthermore, the Polish accessions contained the lowest levels of tannins and trypsin inhibitors. Common vetch seeds may be valuable protein feed/food stuff, but their nutritional usefulness is limited by the presence of anti-nutritional factors, especially HCN, which is difficult to remove. Further selection in this direction may be postulated.

## 1. Introduction

Many crops considered neglected on a global scale may be important at a country or local level and can be used as a supplement to well-balanced diets [1,2]. They include a few species of grain legumes, for example grasspea (*Lathyrus sativus* L.) [3,4,5] and common vetch (*Vicia sativa* L.), the latter of which is the research object of this study. Common vetch is an important legume cultivated for feed grain and forage in Mediterranean and central Asian regions [2,6,7].

Data collected by the Food and Agriculture Organization (FAO) in 2018 showed that, globally, the area harvested and the production of vetch were about 0.541 million ha and 0.934 million tons, respectively [8]. In European Union countries, common vetch occupies 0.122 million ha and the production of vetch is about 0.161 million tons. According to the Official Journal of the European Union [9], the common EU catalogue comprises 128 varieties of common vetch, originating mainly from Spain, Italy, and France. Progress in common vetch breeding in the agronomic terms of limitation of plant susceptibility to lodging and abiotic stresses (drought, diseases, and pests) and grain yields should be related to the ultimate goal of the breeder to make vetch more competitive than other main species of grain legumes.

Little information about this plant, originating from the countries of east and east-central Europe, is available. The agronomic research in common vetch should be simultaneously supported by nutritional investigations in which not only the contents of basal nutrients but also the amino acid (AA) composition, fatty acid (FA) profile, minerals, as well as the level of anti-nutritional factors (ANFs) should be taken into consideration. The ANFs contained in common vetch grain are characterised by different physicochemical properties. Some of them (cyanogenic glycosides, which are the most dangerous toxins, as reported by Collins et al. [10] and Enneking [11]) can dilute in water and can be removed from grain by steeping of the splits in water [12]. Tannins concentrated in seeds can be removed by dehulling, whereas antitrypsin, i.e., a thermolabile proteinase inhibitor, can be inactivated in a thermal treatment [13]. *Vicia sativa* grains may contain HCN [14], either free or in the form of cyanogenic glycosides. Hydrocyanic acid is a dangerous, rapidly acting poison for animals and humans interfering with oxygen use at the cellular level and inhibiting cytochrome oxidase activity [15].

The aforementioned manipulations targeted at making these seeds nutritionally useful are effective but costly and time consuming. It is, therefore, advisable to continue research on breeding of common vetch varieties that are valuable from an agronomic point of view and at the same time rich in protein but containing reduced levels of ANFs. Many world and European gene banks have collected numerous common vetch accessions, representing different geographical origins from all over the world. These accessions create a rich source of unique traits, which can be used as initial material not only for basic research but first of all for breeding purposes. The genetic diversity of the *Vicia* genus, likewise in the *Lathyrus* genus [16,17], is also of considerable importance. In connection with the rapid climatic changes observed for many years, the genetic variation of agricultural traits (expressed by plant adaptation in terms of biotic and abiotic stresses in a changing environment) is highly important. A survey of the diversity of accessions originating from east-, south-, and west-central Europe seems to be of significant importance. It can promote the introduction of the most promising lines from these regions, taking into consideration not only the phenotypic and morphological features but also the nutritional properties of their seeds.

Accession materials showing the different origins of vetch (European countries with different climate-environmental zones) are reflected in the magnitude of the variability of traits that are important for the agricultural and mainly nutritional application. Therefore, the aim of the study was to determine the correlation between the origin of vetch seeds (region, country) and their phenotypic and morphological traits as well as nutritional value. The assessment was based on field studies of accessions originating from European regions and countries grown in the same soil-climate conditions and determination of the content of nutrients and some ANFs in the seeds of common vetch (*Vicia sativa* L.) as well as the variation of their phenotypic and morphological traits.

## 2. Materials and Methods

### 2.1. Plant Material and Study Site

The study was carried out using 44 accessions of common vetch, whose seeds originated from different European countries and were grown in one locality in Poland. Four accessions originated from Germany, Russia, Ukraine, and Hungary, three from the Czech Republic, 19 from the Slovak Republic, and six from Poland. The plant material, except for the Polish accessions, was obtained from the gene bank in Gatersleben (Germany) and Piescany (Slovakia). The accessions represented countries of the following European regions: east and east-central Europe (Russia, Ukraine, and Poland), south-central Europe (Czech Republic, Slovak Republic, and Hungary), and west-central Europe (Germany).

The three-year field study was conducted from 2010 to 2012 on the Experimental Field of the Institute of Plant Genetics in Cerekwica, Poland (51°55′ N, 17°21′ E). The seeds were sown (5th April) in experimental plots (1.5 × 3 m, no edge rows) with 20 cm spacing in rows, 40 cm apart. The plants were grown on brown soil representing the light soil type suitable for potatoes, oats, white lupine, and triticale. Immediately after sowing and before seedling emergence, an Afalon herbicide was applied on the plots in a single dose per soil area. No mineral fertilisation was used during the plant growth. The precipitation in 2010, 2011, and 2012 reached a level of 409, 340, and 430 mm, respectively, from March to the end of September. With only 340 mm of rainfall during this period, 2011 was considered dry.

### 2.2. Analysis of Phenotypic and Morphological Traits of Vetch Accessions

Throughout the vegetation period, the morphological description of plants of the particular accessions was carried out. The plant growth habit was recorded at the onset of the flowering period and the flowering time was expressed as the number of days from sowing to the first flower opening. The flower colour was assessed on the dorsal petal of freshly opened flowers. Plant height was measured at physiological maturity from the ground to the top of the longest branch. After the harvest, the number of pods per plant, pod length, and the number of seeds per pod were determined. The number of pods per plant was evaluated in five randomly selected plants per plot. The pod length and number of seeds per pod were determined in mature pods (10 per plant). Mature seeds were used for estimation of the seed coat colour. The thousand seed weight (TSW) was determined as well (4 × 1000 seeds for repetition).

### 2.3. Chemical Analyses of Vetch Seeds

The content of dry matter and basic nutrients in ground seed samples (250 g of each accession) was determined according to standard AOAC [18] procedures. Nitrogen-free extract content was estimated by the difference. Fibre fractions: neutral-detergent (NDF) and acidic-detergent fibre (ADF) were assayed according to the method proposed by Goering and Van Soest [19]. The contents of amino acids were determined using a Sykam Amino Acid Analyser (Laserchrom HPLC Laboratories Ltd. Inc., Rochester, UK). Prior to analysis, the samples were hydrolysed with 6 N HCl at 110 °C for 24 h. Methionine and cysteine were analysed as methionine sulfone and cysteic acid, respectively, after cold performic acid oxidation overnight before the hydrolysis. Tryptophan was determined after NaOH hydrolysis at 110 °C for 22 h. The average essential amino acid index (EAAI) was calculated according to Oser [20]. The fatty acid composition was determined using the gas chromatography method on a Varian CP-3800 chromatograph (Varian Inc., Palo Alto, CA, USA) after conversion of fats to fatty acid methyl esters (FAME) according to the AOAC method [21]. The chromatograph operating conditions for fatty acid separation were as follows: capillary column CP WAX 52CB DF 0.25 mm of 60 m length, gas carrier-helium, flow rate—1.4 mL min^−1^, column temperature 120 °C gradually increasing by 20 °C per minute, determination time—127 min, feeder temperature—160 °C, detector temperature—160 °C, other gases—hydrogen and oxygen. The determinations were based on a Supelco 37-Component Fame Mix template (Sigma-Aldrich Poznań, Poznań, Poland). The results were expressed as the proportion of individual fatty acids in the total value of fatty acids taken as 100%. The contents of the minerals (except phosphorus) were determined in an ASA SOLAR 939 (Unicam, Cambridge, UK) flame spectrophotometer according to PN–EN ISO 6869 [22], whereas the phosphorus content was assessed with the atomic absorption spectrometry technique at 400 nm according to AOAC [18]. The level of polyphenolic compounds (tannins) was estimated using the method described by Waniska et al. [23] and trypsin inhibitors (TIU) were analysed in accordance with PN-EN ISO 14,902 [24]. Hydrogen cyanide (HCN) was determined according to Makkar et al. [25].

### 2.4. Statistical Analysis

Means were calculated for the quantitative phenotypic and morphological characteristics as well as the contents of nutrients and anti-nutritional factors in the common vetch seeds of the particular accessions originating from different countries and European regions. Chemical analyses were performed in triplicate. The normality of data and homogeneity of variances were tested using the Shapiro-Wilk and Brown-Forsythe tests, respectively. A Box-Cox or log transformation was used to help normalise data that did not meet these assumptions. The Welch F-test correction was used when the data did not meet the assumption of homogeneity of variance. The data obtained were analysed using ANOVA tests of the main effects with the country and region of origin as an independent variable or the non-parametric Kruskal-Wallis test (a non-parametric equivalent of one-way analysis of variance). The response of the variables showed no interaction with the year. Hence, that data were averaged for all three years. Detailed comparisons between the means were conducted using the post hoc Duncan or Dunn test. All statements of significance were based on the 0.05 probability levels. All the data were analysed with the Statistica software version 10.0.

## 3. Results

### 3.1. Phenotypic and Morphological Traits of Vicia sativa L. Accessions

The plant height in the majority of accessions ranged from 33.5 to 49 cm, and an evident relationship with their geographical origin was observed (Table 1). Most of them represented spreading and semi-erect habits. The plant accessions from Slovakia, Ukraine, and Hungary exhibited the greatest height, in comparison with those from Poland (*p* < 0.0001). The plant height differed significantly between the regions of origin. Plants originating from the south-central European accessions were higher than those from east and east-central Europe (47.6 vs. 39.9) (*p* < 0.0001). Irrespective of their origin, the onset of flowering of these plants was noted in the range from 66.6 to 72.8 days (*p* > 0.05).

In terms of the colour of flowers, all accessions represented three easily distinguishable types of inflorescences: the majority (35 accessions) produced blue/violet to purple flowers, eight accessions (mainly from Poland) had white flowers, and one accession had pink flowers (Tables S1–S3). The yield-contributing parameters were expressed by values of the number of pods per plant, pod length, number of seeds per pod, and TSW (Table 1). In the case of the number of pods per plant (42.9–69.8) and the pod length (4.8–5.4 cm), no evident connection with their geographical origin was observed (*p* > 0.05). The seeds were round (somewhat flattened) and smooth. The highest number of seeds per pod was exhibited by the accessions from Poland and Ukraine, whereas the lowest number was determined in the plants from Germany (*p* < 0.0001). The average number of seeds in a pod was seven in the accessions from east- and east-central Europe. This number was markedly lower in the plants from west-central Europe (5.9) and south-central Europe (6.5) (*p* < 0.0001). The heaviest seeds were found in the west-central European accessions (average 58 g TSW). The accessions from south-central Europe had smaller seeds (50.2 g TSW), and the smallest ones with average TSW of 41.3 g were noted in the east and east-central European accessions (*p* < 0.001). The highest thousand seed weight was recorded in the German and Slovak accessions, whereas the lowest value was noted for the Polish accessions (*p* = 0.015). The accessions differed in the seed coat colour (electronic Supplementary Material). Some of them had seeds with a lighter colour (grey to slightly green and bright to slightly yellow), while others were strongly coloured with a visible different level of tint intensity (bright brown, brown, dark brown, or black).

### 3.2. Proximate Nutrient Composition and Detergent Fibre Content in Vetch Seeds

The contents of nutrients and anti-nutritional factors in the seeds (dry matter (DM) basis) of vetch accessions originating from different European countries are shown in Table 2. The dry matter of seeds ranged from 911 g (German accessions) to 915 g (Polish accessions), the content of crude ash ranged from 38.6 to 39.6 g kg^−1^, and the amount of crude fibre ranged from 35.7 g (Hungarian accessions) to 38 g (Ukrainian accessions). No relationship with the plant origin was observed (*p* > 0.05). The average content of crude protein in all the accessions was 342 g kg^−1^. The highest protein levels were exhibited by the seeds of the Slovak accessions (average 358 g), whereas the lowest value of the parameter was noted in the Russian and German (324 g) lines (*p* = 0.021). The seeds of the south-central European accessions were characterised by the highest protein content, in comparison with those from the west-central European lines (*p* = 0.036). Considerable differences in the content of ether extract (EE) and nitrogen free extract (NFE) were found between the accessions of different origins as well. The lowest content of EE in seeds was determined in the German and Czech accessions and the highest level was noted in the Polish accessions (*p* = 0.024). There were also differences in the EE contents in vetch seeds originating from the different European regions (*p* = 0.017). The Russian, German, Hungarian, and Polish accessions exhibited a higher level of NFE in their seeds than that in the Slovak lines (*p* = 0.038). The highest content of NFE was determined in the seeds from west-central Europe, whereas its lowest value was noted in the south-central European accessions (*p* = 0.044). The average contents of the NDF and ADF fractions in 1 kg of grain DM were 121 and 69.8 g, respectively, and there were no considerable differences between accessions originating from the different European regions or countries (*p* > 0.05). 

### 3.3. Mineral Composition in Vetch Seeds

There were some differences in the copper, zinc, and iron contents between the seeds from the particular countries (Table 2). The seeds of the accessions from Ukraine and Poland had the highest content of copper, whereas the lowest level of this element was determined in the seeds of the accessions from Russia and Hungary (*p* = 0.045). The highest content of zinc was detected in the seeds of the Slovak, Polish, Czech, and German accessions, compared with that in the Russian accessions (*p* = 0.035). The iron content in the seeds originating from the Czech, Polish, Ukrainian, and Slovak accessions was higher than in the German and Russian accessions (*p* = 0.018). The seeds of the East, East-Central, and South-Central European lines contained more (*p* = 0.024) iron than those originating from west-central Europe.

### 3.4. Anti-Nutritional Factors in Vetch Seeds

The contents of some ANFs in the seeds (DM basis) of the studied accessions are shown in Table 2. The average level of tannins was 7.9 mg kg^−1^. Significant differences (*p* = 0.029) at a level of circa 1.6 mg kg^−1^ in the tannin content were noted between the seeds of the Russian (8.3 mg kg^−1^) and Slovak accessions (8.4 mg kg^−1^) and the Polish seeds (6.8 mg kg^−1^). There were also differences in the tannin content between the analysed regions. The highest level of this ANF was determined in the seeds of the south-central European accessions, in comparison with the east and east-central European accessions (*p* = 0.042). The lowest TIU level was exhibited by the German and Hungarian accessions (2.1 mg kg^−1^) and the Polish line (2.7 mg kg^−1^). The highest value of this parameter was noted in the case of the vetch seeds from the Ukrainian and Russian accessions (4.4 and 4.3 mg kg^−1^, respectively), with statistically confirmed differences (*p* = 0.036). The seeds of the accessions originating from east and east-central Europe had the highest level of TIU, in comparison with the west-central and south-central European accessions (*p* = 0.028). Considerable differences in the HCN content were noted between the seeds of the accessions from the different countries of Europe, with its highest level recorded in the Russian and German accessions (119 and 126 mg kg^−1^, respectively) and the lowest values noted in the seeds of the Slovak and Polish lines (50 and 66.8 mg kg^−1^, respectively) (*p* = 0.015). The HCN levels in the seeds differed significantly between the regions of plant origin, with the highest values determined in the west-central Europe accessions and the lowest levels noted in south-central Europe (*p* = 0.017).

### 3.5. Essential Amino Acid Content in Vetch Seeds

Table 3 presents the composition of essential amino acids in the seeds of the studied accessions. The highest levels of cysteine were detected in the seed protein in the Russian accessions, whereas the lowest content was noted in the Hungarian, Polish, and Ukrainian lines (*p* = 0.018). This was also reflected in the differences between the analysed regions. The west-central European accessions were characterised by higher (*p* = 0.026) content of cysteine than those from the other assessed regions of Europe. The results indicated the highest content of methionine in the seed protein from the Russian, German and Slovak accessions, compared with the Hungarian lines (*p* = 0.027). No such differences were observed between the regions of origin of the accessions (*p* > 0.05). The highest content of tryptophan was determined in the Russian accessions, and its lowest levels were detected in the protein of the Slovak lines (*p* = 0.012). Similar differences were found in the comparison of the regions of origin. The east, east-central, and west-central European accessions exhibited the highest amounts of tryptophan in the seed protein (*p* = 0.031). Significant differences were noted in the essential amino acid index (EAAI). Its highest value was calculated in the case of protein in the seeds originating from the Russian and German accessions, in comparison with the Hungarian, Slovak, and Czech lines (*p* = 0.042). In terms of the region of origin, its highest value was exhibited by the protein of vetch seeds from west-central Europe (75), and the lowest value (71) was determined for the accessions originating from south-central Europe (*p* = 0.026). 

### 3.6. Fatty Acid Composition in Vetch Seed Fat

Table 4 shows the average fatty acid (FA) composition (expressed as % of total FA) in the vetch seed accessions. The fat from the vetch seeds of the Czech, Hungarian, Polish, and Slovak accessions was characterised by the highest content of polyunsaturated fatty acids (PUFA) (over 67% of total FA), whereas the PUFA lowest levels were recorded in the material from Russia (about 64%) (*p* = 0.043). In turn, the highest share of monounsaturated fatty acids (MUFA) was found in the vetch seed fat from the Russian accessions (over 17% of total FA) (*p* = 0.038). This also yielded differences between the analysed regions of Europe. The seeds of accessions originating from south-central Europe exhibited a lower share of MUFA (about 14%) (*p* = 0.042) than those from the other European regions (over 15%). 

There were some significant differences in the fatty acid contents between the accessions originating from different parts of Europe. The predominant linoleic acid (C18:2 n-6) constituted on average 53% of total FA, with higher contents in the accessions from Czechia and Hungary (about 55% and 54%, respectively) and the lowest level in the seed fat from the Russian accessions (about 51%) (*p* = 0.017). Another polyunsaturated fatty acid with the highest proportion in the vetch seed fat was linolenic acid (C18:3 n-3), on average accounting for 13.5% of total FA, with higher contents in the Slovak and Polish accessions (over 14%) and the lowest level in the seed fat from the Russian and Czech accessions (about 12 and 13%, respectively) (*p* = 0.025). The most favourable n-6/n-3 proportion was found in the fat from the Slovak and Polish seeds (*p* = 0.026). The vetch seed fat also exhibited high content of oleic acid (C18:1 n-9), which represented on average 13.6% of total FA. The highest levels of this monounsaturated fatty acid were determined in the seeds of the Russian accessions (about 16%), whereas the Czech, Slovak, and Polish lines exhibited its lowest content (*p* = 0.035). In terms of the region of origin, the highest amounts of oleic acid were detected in the seeds from the east and east-central European accessions (over 14%), compared with those from south-central Europe (below 13%) (*p* = 0.031). Lignoceric acid was the only saturated fatty acid with different contents depending on the origin of the accessions. Its lowest levels were determined in the fat from the seeds originating from the Czech, Slovak, and German lines, and the highest content of this acid was exhibited by the Russian lines (*p* = 0.015). In terms of the region of origin, the highest content of lignoceric acid was detected in the fat from the seeds of the east and east-central European lines, in comparison with the other assessed regions of Europe (*p* = 0.024).

## 4. Discussion

Common vetch is a food, feed, or fodder crop belonging to the *Leguminosae* family and *Vicieae* tribe. Its spreading and seldom semi-erect habit make vetch very sensitive to lodging [7]. The plant height has been found to vary greatly and may reach over 1 m [26]. Common vetch has very high water requirements [27]. The drought in April and May that occurred during the study period accelerated the onset of plant flowering. The same effect was observed by Jasińska and Kotecki [26], who reported on average 60–70 days to the first flowering, similar to our results. Among the accessions examined in the present study, forms with an earlier (67 d-Czech accession) or later onset of flowering (73 d-Hungarian and Slovak accessions) can be distinguished.

As reported by Chowdhury et al. [28], purple and white are the typical flower colours of vetch, whereas Milczak [29] described the colour of flowers in this species as either blue or white and Jasińska and Kotecki [26] observed violet-red and violet-blue flowers. The blue-violet to purple colour of flowers was predominant in the analysed accessions; however, similar to Chowdhury et al. [28], some white flowering vetch accessions were also found.

In terms of the yielding parameters, the accessions from west-central Europe differed from those representing the other European geographical regions. The differences were particularly visible in comparison with the Polish accessions, characterised by very short plants, the highest number of pods per plant, and the lowest TSW of all the examined accessions. The low TSW in the Polish lines was similar to small-seeded forms originating from south Asia with a TSW value of about 30 g described by Sharma and Kalia [30]. The broad range of variation of yield-contributing traits of common vetch was indicated by Silezin and Szwed-Urbaś [31] in their study on Polish vetch material.

Common vetch seeds have the potential to be a valuable protein source [10]. The average content of protein in the studied accessions (313 g in 1 kg of natural grains) was similar to that found in the seeds of 23 *Vicia sativa* lines analysed by Aletor et al. [32] but higher than the values reported in some other papers. In the study conducted by Uzun et al. [33], where 388 common vetch accessions grown in Turkey were analysed, the average CP level in natural matter of grains was equal to only 245 g kg^−1^. An even lower level of this nutrient (230 g kg^−1^) was determined in *Vicia sativa* grains cultivated in India [30]. Taking into consideration the accessions analysed in the present study, the lowest content of CP was detected in the Russian and German accessions. At the same time, the seeds of the German accessions were characterised by the highest TSW. This corresponds with the results obtained by Uzun et al. [33], who observed a significant decrease in the protein content in vetch seeds with an increase in their size.

The results showed a lower amount of iron in the vetch seed accessions originating from west-central Europe than in those from the other European regions. This feature can be associated with differences in the seed size between the accessions of different origins [33].

The quality of vetch protein is rather low, mainly because of the low content of sulphur-containing acids (methionine, cysteine), with regard to the requirements of monogastric animals [34]. With reference to the relevant norms [34,35], methionine contained in the protein of vetch grains covers only half of growing pig and broiler demands. The amino acid (AA) composition of vetch protein in the investigated accessions originating from the different countries was highly similar. However, the cysteine level was higher in the protein of the west-central Europe lines in comparison with the other European regions. Fat contained in common vetch grains does not play an important role as an energetic nutrient, as its content is usually below 1%. Despite its low concentration but thanks to the valuable composition of its fatty acids, the presence of fat in vetch seeds is very important when they serve as feed or food. Vetch grain fat is rich in linoleic (C18:2 n-6) and linolenic (C18:3 n-3) acids. In the accessions evaluated in this study, linoleic acid represented about 53% of total FA, and linolenic acid was present at a relatively high level (on average over 13% of total FA). Additionally, the n-6/n-3 FA proportion (4 in average) was adequate to animal or human requirements [36]. The investigations of the FA composition in *Vicia sativa* grains are rather scarce in the available literature. The results obtained in this study differ fundamentally from those reported by Akpinar et al. [37], who found linolenic and linoleic acid contents in total FA of vetch seeds to be as low as only 4.3 and 2.0%, respectively. A more similar FA composition was determined in the grains of some *Vicia* species by Kökten et al. [38].

Regarding grasspea, Desphande and Campbell [39] found that the level of condensed tannins in the seeds was highly correlated with the intensity of the seed colour, and a white colour of seeds was associated with white flowers. The results of the present work confirm this correlation in the vetch seeds. Among the accessions originating from the different countries, the lowest (*p* < 0.05) concentration of tannins was noted in the Polish accessions, with their white flowers and bright seed coat, while the highest level of this ANF was determined in the seeds of the Slovak accessions, which had mainly a brown to black seed coat.

The contents of HCN in the seeds of the examined accessions correspond with those reported by Hanelt and Tschiersch [14], who investigated 228 samples of different *Vicia sativa* accessions. They detected from 0 up to 304 mg HCN per kg of seed. With only 0.25 mg HCN per kg per day tolerance, ruminants are more susceptible to poisoning by cyanogenic plants than monogastric animals [40]. As shown in a study carried out by Manzano et al. [41] on growing pigs, the level of 0.8 mg HCN equivalent per kg body weight per day may be defined as NOAEL (no-observed-adverse-effect level) [42]. A similar NOAEL value was reported by Jackson [43] in an experiment on miniature pigs treated with KCN for 24 weeks. Assuming a daily feed intake of 2.5 kg DM per 100 kg body weight of growing pigs [35], i.e., 25 g DM per 1 kg BW, the NOAEL of HCN is equal to 32 mg kg^−1^ DM. Therefore, the average concentration of HCN in the vetch seeds of the accessions originating from the majority of the studied countries exceeded the tolerance level of this ANF. The highest concentration of HCN was determined in the German and Russian accessions. Taking into consideration the content of HCN in these accessions, the allowable proportion of vetch seeds should rather not exceed 20% in monogastric animal diets.

## 5. Conclusions

The present results of the assessment of the phenotypic and morphological traits, nutrient content, and the level of anti-nutritional factors in vetch seeds demonstrate a clear relationship between these parameters and plant origin. The accessions from west-central Europe flowered about three days earlier than those coming from south-central Europe. They also had a significantly lower number of seeds per pod but, at the same time, the highest average thousand seed weight, with the smallest grains of in the Polish accession. The vetch lines from south-central Europe contained the highest level of crude protein in comparison with those from west-central Europe (353 vs. 324 g kg^−1^, respectively). However, their essential amino acid index was the lowest (71 vs. 75, respectively). The analysed lines differed in the concentration of the examined ANFs. The highest HCN level was exhibited by the German and Russian accessions (126 and 119 mg kg^−1^, respectively), and the lowest value was noted in the Slovak and Polish lines (50 and 67 mg kg^−1^, respectively). The Polish accessions contained the lowest level of tannins and trypsin inhibitors. Common vetch seeds may be valuable protein feed/food stuff, but their nutritional usefulness is limited by the presence of anti-nutritional factors, especially hydrogen cyanide, which is difficult to remove. Further studies in this direction may be postulated.

## Figures and Tables

**Table 1:** Average quantitative phenotypic and morphological traits of *Vicia sativa* L. accessions originating from different European regions and countries.

Accessions	Number of Accessions	Traits					
		Plant Height, cm	Days to First Flowering	Number of Pods Per Plant	Pod Length, cm	Number of Seeds Per Pod	Thousand Seed Weight, g
Average for the country of origin							
East and east-central Europe							
Russia	12	42.84 ^a,b^	70.67	60.82	5.36	6.59 ^ab^	43.50 ^a,b^
Ukraine	12	46.53 ^a^	70.83	67.72	5.24	7.12 ^a^	44.55 ^a,b^
Poland	18	33.47 ^b^	70.56	65.13	5.03	7.18 ^a^	37.54 ^b^
South-central Europe							
Czechia	9	40.52 ^a,b^	66.56	67.47	5.36	6.80 ^a,b^	46.40 ^a,b^
Slovakia	57	48.99 ^a^	72.77	69.84	5.30	6.43 ^a,b^	50.97 ^a^
Hungary	12	46.14 ^a^	72.83	64.98	5.06	6.31 ^a,b^	49.29 ^a,b^
West-central Europe							
Germany	12	41.06 ^a,b^	69.50	42.90	4.82	5.86 ^c^	58.00 ^a^
Average for the region of origin							
East and east-central Europe	42	39.88 ^b^	70.67	64.64	5.19	6.99 ^a^	41.25 ^b^
South-central Europe	78	47.57 ^a^	72.06	68.82	5.27	6.45 ^b^	50.19 ^a^
West-central Europe	12	41.06 ^a,b^	69.50	42.90	4.82	5.86 ^c^	58.00 ^a^
Overall mean for the accessions	132	44.53	71.39	65.13	5.20	6.57	48.05
Standard deviation		10.66	5.83	23.71	0.60	0.90	12.50
Source of variation in ANOVA							
Country *p* value		<0.0001	0.053	0.054	0.081	<0.0001	0.015
Region *p* value		<0.0001	0.284	0.069	0.057	<0.0001	<0.001

Note: ^a–c^—values in a column with different letters are significantly different between the country or region of origin (*p* < 0.05).

**Table 2:** Content of proximate nutrients, detergent fibre, some minerals, and anti-nutritional factors in *Vicia sativa* seeds (in 1 kg dry matter (DM) basal seeds).

Accessions	Proximate Nutrients, g						Detergent Fibre, g		Minerals									Anti-Nutritional Factors (ANFs), mg		
	DM	Crude Ash	Crude Protein	Ether Extract (EE)	Crude Fiber	Nitrogen Free Extract (NFE)	Neutral-Detergent (NDF)	Acidic-Detergent Fibre (ADF)	Ca, g	P, g	Mg, g	K, g	Na, g	Cu, mg	Zn, mg	Fe, mg	Mn, mg	Tannins	Trypsin Inhibitors (TIU)	Hydrogen Cyanide (HCN)
Average for the country of origin																				
East and east-central Europe																				
Russia	914.63	39.57	323.57 ^b^	6.93 ^a,b^	37.60	534.27 ^a^	116.27	69.80	2.16	4.69	1.65	12.00	1.53	4.71 ^b^	35.81 ^b^	55.33 ^b^	11.68	8.33 ^a^	4.33 ^a^	118.61 ^a^
Ukraine	912.23	39.13	337.30 ^a,b^	6.77 ^a,b^	37.97	517.87 ^a,b^	121.23	68.63	2.28	4.74	1.60	12.15	1.73	5.48 ^a^	38.47 ^a,b^	61.54 ^a^	11.39	8.25 ^a,b^	4.37 ^a^	97.18 ^a,b^
Poland	915.20	38.27	335.60 ^a,b^	7.43 ^a^	37.47	523.23 ^a^	121.73	69.63	2.03	4.65	1.49	11.66	1.46	5.44 ^a^	43.84 ^a^	63.59 ^a^	12.79	6.76 ^c^	2.67 ^c^	66.77 ^d,c^
South-central Europe																				
Czechia	913.03	37.83	348.50 ^a,b^	6.37 ^b^	37.37	510.03 ^a,b^	121.13	71.40	1.89	4.75	1.54	11.93	1.45	5.18 ^a,b^	43.43 ^a^	63.97 ^a^	12.63	7.69 ^b^	3.59 ^b^	73.99 ^b–d^
Slovakia	912.47	38.57	358.37 ^a^	6.53 ^a,b^	36.30	499.33 ^b^	124.90	70.23	1.89	4.80	1.54	12.37	1.46	5.20 ^a,b^	43.94 ^a^	60.74 ^a^	12.60	8.36 ^a^	3.33 ^b^	50.34 ^d^
Hungary	913.37	38.40	335.83 ^a,b^	6.57 ^a,b^	35.67	523.43 ^a^	116.33	69.50	2.03	4.26	1.60	12.21	1.37	4.76 ^b^	41.87 ^a,b^	55.69 ^a,b^	11.76	7.93 ^b^	2.07 ^c^	110.03 ^a,b^
West-central Europe																				
Germany	911.17	38.40	324.17 ^b^	6.03 ^b^	36.27	533.63 ^a^	117.47	70.13	2.23	4.32	1.59	12.65	1.54	5.21 ^a,b^	42.62 ^a^	55.27 ^b^	12.71	7.89 ^b^	2.10 ^c^	126.46 ^a^
Average for the region of origin																				
East and east-central Europe	914.19	38.88	332.64 ^a,b^	7.13 ^a^	37.65	524.83 ^a,b^	120.01	69.39	2.14	4.69	1.57	11.90	1.56	5.24	40.01	60.64 ^a^	12.07	7.63 ^b^	3.63 ^a^	90.27 ^b^
South-central Europe	912.79	38.44	352.65 ^a^	6.53 ^a,b^	36.37	505.55 ^b^	122.92	70.29	1.91	4.68	1.55	12.24	1.43	5.10	43.44	60.24 ^a^	12.45	8.16 ^a^	3.08 ^b^	66.01 ^c^
West-central Europe	911.17	38.40	324.43 ^b^	6.03 ^b^	36.27	533.63 ^a^	117.47	70.13	2.23	4.32	1.59	12.65	1.54	5.21	42.62	55.27 ^b^	12.71	7.89 ^a,b^	2.09 ^c^	126.46 ^a^
Overall mean for the accessions	913.26	38.60	342.24	6.70	36.81	515.71	121.32	69.84	2.04	4.65	1.56	12.14	1.49	5.16	41.98	59.90	12.33	7.93	3.19	81.14
Standard deviation	8.40	2.60	29.97	0.65	9.47	12.30	14.30	4.76	0.35	0.36	0.16	0.79	0.15	0.49	3.71	5.65	2.42	0.84	0.93	35.63
Source of variation in ANOVA																				
Country *p* value	0.431	0.361	0.021	0.024	0.590	0.038	0.189	0.637	0.474	0.371	0.446	0.256	0.156	0.045	0.035	0.018	0.327	0.029	0.036	0.015
Region *p* value	0.672	0.745	0.036	0.017	0.814	0.044	0.266	0.792	0.409	0.594	0.838	0.423	0.687	0.592	0.119	0.024	0.840	0.042	0.028	0.017

Note: ^a–d^—values in a column with different letters are significantly different among the country or region of origin (*p* < 0.05).

**Table 3:** Average essential amino acid content (g 100 g^−1^ of crude protein) in *Vicia sativa* seeds.

Accessions	Amino Acids											
	Thr	Cys	Met	Val	Ile	Leu	Tyr	Phe	His	Lys	Trp	EAAI
Average for the country of origin												
East and east-central Europe												
Russia	3.48	1.14 ^a^	1.05 ^a^	3.77	2.55	7.02	2.47	3.59	2.49	5.79	3.70 ^a^	75.79 ^a^
Ukraine	3.53	0.93 ^b^	0.95 ^a,b^	3.83	2.50	7.16	2.46	3.63	2.45	5.76	3.23 ^a,b^	73.59 ^a,b^
Poland	3.61	0.88 ^b^	0.88 ^a,b^	3.83	2.49	7.23	2.58	3.70	2.47	5.87	3.19 ^a,b^	74.02 ^a,b^
South-central Europe												
Czechia	3.39	0.78 ^b^	0.84 ^a,b^	3.68	2.39	6.91	2.40	3.65	2.51	5.69	3.09 ^b^	70.45 ^b^
Slovakia	3.42	1.06 ^a,b^	0.91 ^a^	3.74	2.41	6.96	2.46	3.57	2.49	5.71	2.82 ^c^	72.00 ^b^
Hungary	3.53	0.83 ^b^	0.72 ^b^	3.90	2.54	7.19	2.50	3.64	2.55	5.86	2.94 ^b,c^	71.96 ^b^
West-central Europe												
Germany	3.57	1.04 ^a,b^	0.93 ^a^	3.84	2.56	7.27	2.53	3.61	2.52	5.91	3.30 ^a,b^	75.16 ^a^
Average for the region of origin												
East and east-central Europe	3.55	0.97 ^b^	0.95	3.81	2.51	7.15	2.51	3.65	2.47	5.81	3.34 ^a^	74.47 ^a,b^
South-central Europe	3.44	0.97 ^b^	0.86	3.76	2.43	7.00	2.46	3.60	2.50	5.74	2.88 ^b^	71.47 ^b^
West-central Europe	3.57	1.04 ^a^	0.93	3.84	2.56	7.27	2.53	3.61	2.52	5.91	3.30 ^a^	75.16 ^a^
Overall mean for the accessions	3.50	0.98	0.90	3.79	2.47	7.09	2.49	3.62	2.49	5.78	3.10	73.28
Standard deviation	0.18	0.19	0.20	0.34	0.27	0.33	0.18	0.30	0.22	0.35	0.27	1.94
Source of variation in ANOVA												
Country *p* value	0.680	0.018	0.027	0.433	0.662	0.481	0.365	0.423	0.285	0.362	0.012	0.042
Region *p* value	0.724	0.026	0.184	0.581	0.712	0.562	0.786	0.599	0.631	0.439	0.031	0.026

Note: ^a–c^—values in a column with different letters are significantly different among the country or region of origin (*p* < 0.05).

**Table 4:** Fatty acid composition (% of total fatty acids) in *Vicia sativa* seed fat.

Accessions	Fatty Acids													Fatty Acid Groups			
	C 14:0	C 16:0	C 16:1. n-7	C 18:0	C 18:1 n-9	C 18:1 n-7	C 18:2 n-6	C 18:3 n-3	C 20:0	C 20:1 n-11	C 22:1 n-9	C 24:0	Other	SFA	MUFA	PUFA	n-6/n-3 PUFA Ratio
Average for the country of origin																	
East and east-central Europe																	
Russia	0.44	11.78	0.16	4.00	16.05 ^a^	0.53	51.15 ^b^	12.43 ^b^	1.77	0.53	0.16	0.37 ^a^	0.64	18.36	17.42 ^a^	63.58 ^b^	4.13 ^a^
Ukraine	0.41	11.30	0.22	3.85	14.28 ^a,b^	0.50	52.14 ^a,b^	13.81 ^a,b^	1.94	0.48	0.15	0.27 ^a,b^	0.63	17.76	15.66 ^a,b^	65.95 ^a,b^	3.78 ^a,b^
Poland	0.50	11.68	0.27	3.70	13.08 ^b^	0.53	53.03 ^a,b^	14.14 ^a^	1.69	0.46	0.16	0.22 ^a,b^	0.54	17.79	14.49 ^b^	67.17 ^a^	3.76 ^b^
South-central Europe																	
Czechia	0.52	11.88	0.16	3.61	12.40 ^b^	0.58	54.60 ^a^	12.99 ^b^	1.51	0.53	0.37	0.14 ^b^	0.71	17.66	14.03 ^b^	67.59 ^a^	4.23 ^a^
Slovakia	0.54	11.67	0.17	3.69	12.59 ^b^	0.54	52.88 ^a,b^	14.29 ^a^	2.08	0.53	0.19	0.15 ^b^	0.67	18.14	14.01 ^b^	67.17 ^a^	3.70 ^b^
Hungary	0.46	11.74	0.21	3.64	13.16 ^a,b^	0.52	54.02 ^a^	13.29 ^a,b^	1.58	0.43	0.12	0.22 ^a,b^	0.61	17.64	14.44 ^b^	67.31 ^a^	4.07 ^a^
West-central Europe																	
Germany	0.43	11.11	0.19	3.96	13.73 ^a,b^	0.55	53.17 ^a,b^	13.19 ^a,b^	2.06	0.53	0.18	0.15 ^b^	0.75	17.71	15.18 ^a,b^	66.36 ^a,b^	4.05 ^a^
Average for the region of origin																	
East and east-central Europe	0.46	11.60	0.22	3.82	14.27 ^a^	0.52	52.24	13.56	1.79	0.48	0.15	0.28 ^a^	0.60	17.95	15.66 ^a^	65.80	3.87
South-central Europe	0.51	11.77	0.18	3.69	12.78 ^b^	0.53	53.61	13.67	1.76	0.48	0.21	0.16 ^b^	0.67	17.87	14.17 ^b^	67.28	3.93
West-central Europe	0.43	11.11	0.19	3.96	13.73 ^a,b^	0.55	53.17	13.19	2.06	0.53	0.18	0.15 ^b^	0.75	17.71	15.18 ^a^	66.36	4.05
Overall mean for the accessions	0.47	11.63	0.20	3.74	13.55	0.53	53.16	13.46	1.75	0.48	0.17	0.23	0.64	17.80	14.94	66.62	3.96
Standard deviation	0.18	0.98	0.11	0.35	1.90	0.18	3.68	1.14	0.71	0.19	0.08	0.12	0.28	1.35	1.41	2.79	0.32
Source of variation in ANOVA																	
Country *p* value	0.194	0.321	0.249	0.227	0.035	0.376	0.017	0.025	0.090	0.190	0.277	0.015	0.315	0.663	0.038	0.043	0.026
Region *p* value	0.488	0.623	0.425	0.542	0.031	0.429	0.148	0.276	0.073	0.301	0.454	0.024	0.627	0.734	0.042	0.183	0.078

Note: ^a,b^—values in a column with different letters are significantly different among the country or region of origin (*p* < 0.05).

## Data Availability

Not applicable.

## Supplementary Materials

The following are available online at https://www.mdpi.com/2076-2615/11/1/44/s1, Table S1: Origin and estimation of morphological traits of *Vicia sativa* L. accessions–year 2010, Table S2: Origin and estimation of morphological traits of *Vicia sativa* L. accessions–year 2011, Table S3: Origin and estimation of morphological traits of *Vicia sativa* L. accessions–year 2012.

## Abbreviations

AA, amino acid; ADF, acid detergent fibre; ADL, acid detergent lignin; ANFs, anti-nutritional factors; DM, dry matter; EAAI, essential amino acid index; EE, ether extract; FA, fatty acid; HCN, hydrogen cyanide; MUFA, monounsaturated fatty acids; NDF, neutral detergent fibre; NFE, nitrogen free extract; PUFA, polyunsaturated fatty acids; SFA, saturated fatty acids; TIU, trypsin inhibitors; TSW, thousand seed weight.
