# Supplementary material for: Nutritional and Anti-Nutritional Factors in Vicia sativa L. Seeds and the Variability of Phenotypic and Morphological Characteristics of Some Vetch Accessions Cultivated in European Countries
Source: Animals (Basel). 2020 Dec 28;11(1):44. doi: 10.3390/ani11010044 (PMC7824106; doi:10.3390/ani11010044)
Supplement: Supplementary file 1 [file animals-11-00044-s001.pdf]

ELECTRONIC SUPPLEMENTARY MATERIAL OF THE ARTICLE:

Nutritional and Anti-Nutritional Factors in *Vicia sativa* L. Seeds and the Variability of Phenotypic and Morphological Characteristics of Some Vetch Accessions Cultivated in European Countries

Supplementary Table S1. Origin and estimation of morphological traits of *Vicia sativa* L. accessions – year 2010.

Accessions	Country of origin	Flower colour	Seed coat colour
VSAT 1	Germany	Blue/violet	Dark
VSAT 2	Germany	Blue/violet	Slightly brown
VSAT 3	Germany	Blue/violet	Slightly brown
VSAT 4	Germany	Blue/violet	Slightly brown to gray
VSAT 5	Russia	Blue/violet	Bright to cream
VSAT 6	Russia	White	Bright to slightly brown
VSAT 7	Russia	Blue/violet	Bright to slightly green
VSAT 8	Russia	Blue/violet	Dark to black
VSAT 9	Ukraine	Blue/violet	Dark with pigmentation
VSAT 10	Ukraine	Blue/violet	Deep dark to black
VSAT 11	Ukraine	Blue/violet	Slightly brown
VSAT 12	Ukraine	Blue/violet	Cream to slightly green
VSAT 13	Czech	Blue/violet	Slightly brown to cherry/red
VSAT 14	Czech	Blue/violet	Light brown
VSAT 15	Czech	Blue/violet	Light brown
VSAT 16	Hungary	Blue/violet	Light brown
VSAT 17	Hungary	Blue/violet	Dark brown
VSAT 18	Hungary	Blue/violet	Dark brown
VSAT 19	Hungary	White	Bright brown
VSAT 20	Slovakia	Blue/violet	Light brown to dark brown
VSAT 21	Slovakia	Blue/violet	Bright to gray
VSAT 22	Slovakia	Blue/violet	Bright to slightly green
VSAT 23	Slovakia	Blue/violet	Dark brown
VSAT 24	Slovakia	Blue/violet	Bright brown to gray
VSAT 25	Slovakia	Blue/violet	Dark brown to black
VSAT 26	Slovakia	Pink	Dark brown to black
VSAT 27	Slovakia	White	Light brown
VSAT 28	Slovakia	Blue/violet	Light brown
VSAT 29	Slovakia	Blue/violet	Light brown
VSAT 30	Slovakia	Blue/violet	Deep dark
VSAT 31	Slovakia	Blue/violet	Dark to black
VSAT 32	Slovakia	Blue/violet	Brown
VSAT 33	Slovakia	Blue/violet	Dark to black
VSAT 34	Slovakia	Blue/violet	Brown
VSAT 35	Slovakia	Blue/violet	Gray to slightly green
VSAT 36	Slovakia	Blue/violet	Dark to black
VSAT 37	Slovakia	Blue/violet	Dark
VSAT 38	Slovakia	Blue/violet	Dark to gray
VSAT 39	Poland	White	Bright to slightly yellow
VSAT 40	Poland	Blue/violet	Bright to slightly yellow
VSAT 41	Poland	White	Gray to slightly green
VSAT 42	Poland	White	Bright to slightly yellow
VSAT 43	Poland	White	Cream to gray
VSAT 44	Poland	White	Bright to slightly yellow

Supplementary Table S2. Origin and estimation of morphological traits of *Vicia sativa* L. accessions – year 2011.

Accessions	Country of origin	Flower colour	Seed coat colour
VSAT 1	Germany	Blue/violet	Dark
VSAT 2	Germany	Blue/violet	Slightly brown
VSAT 3	Germany	Blue/violet	Slightly brown
VSAT 4	Germany	Blue/violet	Slightly brown to gray
VSAT 5	Russia	Blue/violet	Bright to cream
VSAT 6	Russia	White	Bright to slightly brown
VSAT 7	Russia	Blue/violet	Bright to slightly green
VSAT 8	Russia	Blue/violet	Dark to black
VSAT 9	Ukraine	Blue/violet	Dark with pigmentation
VSAT 10	Ukraine	Blue/violet	Deep dark to black
VSAT 11	Ukraine	Blue/violet	Slightly brown
VSAT 12	Ukraine	Blue/violet	Cream to slightly green
VSAT 13	Czech	Blue/violet	Slightly brown to cherry/red
VSAT 14	Czech	Blue/violet	Light brown
VSAT 15	Czech	Blue/violet	Light brown
VSAT 16	Hungary	Blue/violet	Light brown
VSAT 17	Hungary	Blue/violet	Dark brown
VSAT 18	Hungary	Blue/violet	Dark brown
VSAT 19	Hungary	White	Bright brown
VSAT 20	Slovakia	Blue/violet	Light brown to dark brown
VSAT 21	Slovakia	Blue/violet	Bright to gray
VSAT 22	Slovakia	Blue/violet	Bright to slightly green
VSAT 23	Slovakia	Blue/violet	Dark brown
VSAT 24	Slovakia	Blue/violet	Bright brown to gray
VSAT 25	Slovakia	Blue/violet	Dark brown to black
VSAT 26	Slovakia	Pink	Dark brown to black
VSAT 27	Slovakia	White	Light brown
VSAT 28	Slovakia	Blue/violet	Light brown
VSAT 29	Slovakia	Blue/violet	Light brown
VSAT 30	Slovakia	Blue/violet	Deep dark
VSAT 31	Slovakia	Blue/violet	Dark to black
VSAT 32	Slovakia	Blue/violet	Brown
VSAT 33	Slovakia	Blue/violet	Dark to black
VSAT 34	Slovakia	Blue/violet	Brown
VSAT 35	Slovakia	Blue/violet	Gray to slightly green
VSAT 36	Slovakia	Blue/violet	Dark to black
VSAT 37	Slovakia	Blue/violet	Dark
VSAT 38	Slovakia	Blue/violet	Dark to gray
VSAT 39	Poland	White	Bright to slightly yellow
VSAT 40	Poland	Blue/violet	Bright to slightly yellow
VSAT 41	Poland	White	Gray to slightly green
VSAT 42	Poland	White	Bright to slightly yellow
VSAT 43	Poland	White	Cream to gray
VSAT 44	Poland	White	Bright to slightly yellow

Supplementary Table S3. Origin and estimation of morphological traits of *Vicia sativa* L. accessions – year 2012

Accessions	Country of origin	Flower colour	Seed coat colour
VSAT 1	Germany	Blue/violet	Dark
VSAT 2	Germany	Blue/violet	Slightly brown
VSAT 3	Germany	Blue/violet	Slightly brown
VSAT 4	Germany	Blue/violet	Slightly brown to gray
VSAT 5	Russia	Blue/violet	Bright to cream
VSAT 6	Russia	White	Bright to slightly brown
VSAT 7	Russia	Blue/violet	Bright to slightly green
VSAT 8	Russia	Blue/violet	Dark to black
VSAT 9	Ukraine	Blue/violet	Dark with pigmentation
VSAT 10	Ukraine	Blue/violet	Deep dark to black
VSAT 11	Ukraine	Blue/violet	Slightly brown
VSAT 12	Ukraine	Blue/violet	Cream to slightly green
VSAT 13	Czech	Blue/violet	Slightly brown to cherry/red
VSAT 14	Czech	Blue/violet	Light brown
VSAT 15	Czech	Blue/violet	Light brown
VSAT 16	Hungary	Blue/violet	Light brown
VSAT 17	Hungary	Blue/violet	Dark brown
VSAT 18	Hungary	Blue/violet	Dark brown
VSAT 19	Hungary	White	Bright brown
VSAT 20	Slovakia	Blue/violet	Light brown to dark brown
VSAT 21	Slovakia	Blue/violet	Bright to gray
VSAT 22	Slovakia	Blue/violet	Bright to slightly green
VSAT 23	Slovakia	Blue/violet	Dark brown
VSAT 24	Slovakia	Blue/violet	Bright brown to gray
VSAT 25	Slovakia	Blue/violet	Dark brown to black
VSAT 26	Slovakia	Pink	Dark brown to black
VSAT 27	Slovakia	White	Light brown
VSAT 28	Slovakia	Blue/violet	Light brown
VSAT 29	Slovakia	Blue/violet	Light brown
VSAT 30	Slovakia	Blue/violet	Deep dark
VSAT 31	Slovakia	Blue/violet	Dark to black
VSAT 32	Slovakia	Blue/violet	Brown
VSAT 33	Slovakia	Blue/violet	Dark to black
VSAT 34	Slovakia	Blue/violet	Brown
VSAT 35	Slovakia	Blue/violet	Gray to slightly green
VSAT 36	Slovakia	Blue/violet	Dark to black
VSAT 37	Slovakia	Blue/violet	Dark
VSAT 38	Slovakia	Blue/violet	Dark to gray
VSAT 39	Poland	White	Bright to slightly yellow
VSAT 40	Poland	Blue/violet	Bright to slightly yellow
VSAT 41	Poland	White	Gray to slightly green
VSAT 42	Poland	White	Bright to slightly yellow
VSAT 43	Poland	White	Cream to gray
VSAT 44	Poland	White	Bright to slightly yellow
